# Assessing the Economic Impact of Vaccine Availability When Controlling Foot and Mouth Disease Outbreaks

**DOI:** 10.3389/fvets.2018.00047

**Published:** 2018-03-13

**Authors:** Thibaud Porphyre, Karl M. Rich, Harriet K. Auty

**Affiliations:** ^1^Royal (Dick) School of Veterinary Studies, The Roslin Institute, University of Edinburgh, Midlothian, United Kingdom; ^2^Epidemiology Research Unit, Scotland’s Rural College, Inverness, United Kingdom; ^3^East and Southeast Asia Regional Office, International Livestock Research Institute, Hanoi, Vietnam

**Keywords:** foot-and-mouth disease, vaccination, control strategies, benefit-cost analysis, vaccine stocks

## Abstract

Predictive models have been used extensively to assess the likely effectiveness of vaccination policies as part of control measures in the event of a foot and mouth disease (FMD) outbreak. However, the availability of vaccine stocks and the impact of vaccine availability on disease control strategies represent a key uncertainty when assessing potential control strategies. Using an epidemiological, spatially explicit, simulation model in combination with a direct cost calculator, we assessed how vaccine availability constraints may affect the economic benefit of a “vaccination-to-live” strategy during a FMD outbreak in Scotland, when implemented alongside culling of infected premises and dangerous contacts. We investigated the impact of vaccine stock size and restocking delays on epidemiological and economic outcomes. We also assessed delays in the initial decision to vaccinate, maximum daily vaccination capacity, and vaccine efficacy. For scenarios with conditions conducive to large outbreaks, all vaccination strategies perform better than the strategy where only culling is implemented. A stock of 200,000 doses, enough to vaccinate 12% of the Scottish cattle population, would be sufficient to maximize the relative benefits of vaccination, both epidemiologically and economically. However, this generates a wider variation in economic cost than if vaccination is not implemented, making outcomes harder to predict. The probability of direct costs exceeding *£*500 million is reduced when vaccination is used and is steadily reduced further as the size of initial vaccine stock increases. If only a suboptimal quantity of vaccine doses is initially available (100,000 doses), restocking delays of more than 2 weeks rapidly increase the cost of controlling outbreaks. Impacts of low vaccine availability or restocking delays are particularly aggravated by delays in the initial decision to vaccinate, or low vaccine efficacy. Our findings confirm that implementing an emergency vaccination-to-live strategy in addition to the conventional stamping out strategy is economically beneficial in scenarios with conditions conducive to large FMD outbreaks in Scotland. However, the size of the initial vaccine stock available at the start of the outbreak and the interplay with other factors, such as vaccine efficacy and delays in restocking or implementing vaccination, should be considered in making decisions about optimal control strategies for FMD outbreaks.

## 1 Introduction

Foot and mouth disease (FMD) remains a constant threat to the livestock sector of the United Kingdom (UK). The 2001 FMD outbreak in the UK was one of the most costly livestock disease outbreaks reported, generating economic losses of over *£*8 billion (1), while a smaller outbreak in 2007 cost the British livestock sector *£*100 million and the government *£*47 million (2).

Current European and national disease control protocols mandate the culling of all susceptible animals on premises where FMD is identified (“infected premises,” IPs) and on those that have had epidemiological contact with IPs (“dangerous contacts,” DCs) to prevent disease spread, known as “stamping out.” The costs, logistics, and ethics of such a strategy, particularly for large outbreaks, are potentially challenging, however. For instance, in the 2001 FMD outbreaks in the UK, 6 million animals were slaughtered either due to infection or to limit the spread of the disease (1).

Given the challenges associated with a culling strategy, European legislation mandates consideration of vaccination in the event of an FMD outbreak. If vaccination was undertaken in Scotland, an emergency “vaccinate-to-live” policy would be used (i.e., vaccinated animals would not require routine culling after the outbreak), and would be carried out alongside conventional “stamping out” (in this paper referred to as a “cull plus vaccinate-to-live” policy). The rationale of such a strategy is that, while IPs and DCs would still be depopulated, the local increase in immunity would reduce the spread of FMD, and hence reduce the overall number of animals to be culled. Although this approach is widely discussed, it has never been undertaken in the European Union (EU), and many questions still exist regarding its likely benefits.

Recent research to quantify the epidemiological effects of a “cull plus vaccinate-to-live” policy in Scotland found that, in general, the net marginal benefit of such a policy was positive when facing widespread outbreaks, though this varied by regional context (3). While these results suggest an important role for vaccination in the case of large outbreaks, an important policy implication concerns the logistics of a vaccination policy itself. In the case of a large outbreak that affected Scotland and the rest of the UK (and possibly other parts of the EU), it remains an open question as to whether sufficient vaccine stocks and delivery capacity for such stocks could be mobilized adequately to arrest the spread of disease. Indeed, should a delay arise in the middle of a vaccination control campaign, it is not clear *a priori* how that might influence the progression of the outbreak as well as the potential direct costs associated with it. Should capacity constraints in vaccine delivery be significant, these delays could not only undermine the success of a vaccination campaign, but also impose significant costs on scarce veterinary resources.

These issues of capacity constraints have come up in other contexts. (4) and (5) highlighted the importance of vaccine capacity in the context of bird flu. (6) looked at the impact of different FMD control strategies in the context of capacity to administer vaccination, as have other recent STUDIES on simulation approaches to the management of FMD vaccination strategies (7–9). However, no studies have specifically assessed the impact of the dynamics of vaccine stocks and their availability. Here, we focus in particular ON the impacts that vaccine stocks and vaccine delivery delays could have on the evolution and total cost of an outbreak, depending on when these constraints occur. In doing so, we provide improved information to decision makers on how to appropriately plan for contingencies associated with appropriate levels of vaccine stocks.

In this paper, we analyze the potential impact of vaccination constraints on simulated outbreaks of FMD in Scotland. We adapt the epidemiological model developed by Porphyre et al. (3) to consider different scenarios of capacity constraints and their effects on disease evolution and direct costs. As such, we assess not only epidemiological impacts, but also the direct costs associated with the outbreak. Our aim is to provide insights to policymakers on the importance of logistical constraints in making decisions on vaccination, including identifying any potential unintended consequences of adopting vaccination policies. We then suggest measures to help mitigate these challenges.

## 2 Materials and Methods

### 2.1 Modeling Framework

The Warwick FMD model (6, 10–13) was used to simulate the various scenarios of vaccination. This model is a fully stochastic, spatial, farm-based model that was developed and used during the FMD epidemic in 2001 in Great Britain (10). It was later modified to represent the Scottish livestock industry (3). Either in its original formulation or in its Scottish version, this model has been extensively used to investigate the value of specific culling and vaccination strategies with respect to variations in epidemic conditions and control responses (3, 6, 11–13). We restricted our scenarios to FMD virus strains circulating within the cattle and sheep industries. As such, the model is restricted to all farms showing at least one animal susceptible to FMD (cattle or sheep). We assume that farms pass through four epidemiological states: susceptible; infected, but not infectious; infectious; or reported infected and thereby culled. The model assumes that each *i^th^* premises is infected with a daily probability depending on its own susceptibility *S_i_* and on the transmissibility *T_j_* of the surrounding j premises. For the *n* premises involved in the study population, each *i^th^* premises has a daily probability *M_i_* to be infected such that

(1) $$M_{i}=1-\text{exp}(-S_{i}\sum_{j\neq i}^{n}\;T_{j}K(d_{ij}))$$

where *S_i_* and *T_j_* depend on the species (i.e., cattle and sheep) and on the related herd size on premises (14, 15). The component *K*(*d_ij_*) is the so-called “transmission kernel function” and determines the scaling factor on the rate at which infected premises may infect susceptible ones as a function of inter-farm distance *d_ij_*.

In line with previous versions of the model (3, 6, 10, 11), we assumed that all farms are infected for 5 days before becoming infectious, and are infectious for 4 days before being reported with infection. The model further considers that once an initial infected premises (IP) is reported, a national movement ban (NMB) would be put in place. Culling measures on each IP would be implemented within 24 h. In addition to the routine culling of IPs, premises where animals have been in direct contact with infected animals or have, in any way, become exposed to infection, known as dangerous contacts (DCs), are culled within 48 h. Premises defined as DCs are determined based upon both prior infection by an IP and future risk of infection (6). Although we assumed that the FMD virus strain involved in outbreaks would only circulate within the cattle and sheep industry, pig premises may still be subject to slaughter for disease control purposes (3, 6). Once animals at an IP are slaughtered, disinfection procedures are initiated and no transmission events to other premises may occur. Preemptive culling based only on spatial proximity (known as “contiguous culling”) was not considered.

### 2.2 Vaccination and Control Scenarios

In line with the Scottish Government’s FMD contingency plan, if vaccination was to be implemented, we assumed that only cattle would be vaccinated (16) and that vaccinated animals would become immune to infection after 4 days. As in previous work (3, 6), we make the conservative assumption that during this 4-day delay, all cattle are completely susceptible and if infected, the disease progresses in the same way as for non-vaccinated cattle. Unless otherwise stated, we considered that 90% of cattle present on vaccinated farms would become totally immune, while the rest would remain totally susceptible to infection and be able to transmit the virus to farms that were not vaccinated (6). Unless otherwise stated, we assumed that the vaccination campaign would start 14 days after the disease is first detected, allowing the decision to vaccinate to be taken, the doses of vaccine to be received from the appropriate vaccine bank and vaccination teams to be mobilized and actively deployed in the field. Once the decision to vaccinate has been made, vaccination would be implemented within a 10-km-radius buffer around each IP (16) and carried out within the recommended 24 h (17). Vaccination within each ring is performed from the outside in, which corresponds to standard policy (6).

Although the model assumes that a decision to vaccinate will be maintained throughout the outbreak (i.e., as disease spreads to new areas new vaccination zones will be created), the vaccination campaign would depend on the number of doses available. In the situation where the supply of vaccine is large enough, we assumed that the capacity to vaccinate would depend only on the level of human resources available. Here, we assumed that 50 vaccination teams would be mobilized (in line with Scottish Government plans), each of which can vaccinate up to 250 animals per day (18). This corresponds to a maximum of 12,500 animals vaccinated per day. In reality, the size of cattle herds in Scotland ranges from 1 to 6,873 head (in 2011), with a median (interquartile range) of 92 head of cattle (26–213 head). As such, it is unlikely that vaccinating 12,500 animals per day would be achieved, since vaccination teams can only travel to a limited number of cattle farms per day. Therefore, we assumed that, while the fixed daily vaccination capacity was 12,500 animals, a maximum of 125 farms could be vaccinated per day but also explored the impact of this parameter.

We considered a number of different scenarios associated with (i) the availability of vaccine stocks at the beginning of the outbreak and (ii) the capacity to re-order new stocks and the time delay required to obtain them. In the first case, we considered the evolution of FMD outbreaks under a vaccination strategy when the initial stock of vaccine varied between 100,000 and 5 million doses, sufficient to vaccinate 6% to nearly 300% of the 1.68 million head of cattle in Scotland. In the second case, we considered a scenario where an initial stock of 100,000 doses is available and explored the impacts of delays in obtaining new stocks ranging from 2 to 16 weeks. Should capacity constraints in the supply of vaccine be significant, vaccination would be carried out normally until no vaccine doses remain. In reality, however, disease control managers may order a new stock of vaccine from the appropriate vaccine bank when the level of the vaccine stock reaches a threshold. Here, a threshold of 10% and 50% remaining of the initial stock were considered. As delays in the production and delivery of the new supply of vaccine may occur, we further considered that vaccine would be only available several days after the date of the order. Unless otherwise stated, we assumed a restocking delay of 14 days.

There are also uncertainties with regards to the vaccine efficacy, the delay in implementing vaccination and the maximum capacity of vaccinating cattle farms, which may impact on the benefit of re-ordering new stocks when a time delay to obtain them is introduced. We, therefore, evaluated the impact of these constraints in the role played by restocking delays on the evolution of an outbreak. We considered (i) a maximum vaccination capacity of 75, 100, 125, 150, or 165 farms per day, (ii) the implementation of the vaccination strategy at 7, 14, or 21 days post detection of the index case, and (iii) that vaccination confers 70% or 90% immunity.

### 2.3 Model Implementation

For all tested scenarios, 10,000 epidemics were simulated assuming that FMD is introduced in a single susceptible herd and spread silently to four additional herds prior to detection. All initial infected herds are located in the county of Ayrshire, which has a high density of premises and animals, and has been previously identified as an area where there is potential for extensive initial spread, and hence a greater benefit from vaccination if an FMD outbreak occurred (3). For the purpose of this study, each simulation starts with the same set of initial infected herds. It is important to note that while the incursion events begin in Ayrshire, all herds present in mainland Scotland are susceptible to infection in the model.

### 2.4 Quantification of the Direct Costs of an FMD Outbreak

We focused on the operational costs associated with an FMD outbreak occurring in Scotland and independently from the rest of Great Britain (GB). While Scotland is part of an epidemiological unit comprising GB and cross-border disease transmission would occur, management of animal health and disease control are fully devolved to the Scottish Government, meaning that disease control decisions are made independently by Scotland. Operational costs were defined as costs directly related to disease control activities and include, not only the cost of culling and vaccinating livestock, but also, among others, the cost of local movement restrictions and international trade bans. Taken together, the operational costs considered in this study form the overall direct cost of controlling FMD outbreaks and was estimated in 2011 equivalent pounds sterling. Wider economic costs, such as the impact on other rural businesses and tourism, were not considered in this analysis, and indirect costs of market reactions to an outbreak were not included. Table 1 details specific cost elements considered in the estimate of the direct cost, and whether they are incurred by the government (in this case Scottish Government) or by the livestock industry.

**Table 1:** Breakdown of economic costs by group.

	Disease control cost	Compensated cost	Non-compensated cost
Government	Management cost Identifying IPs and DCs Depopulation Preliminary C&D Surveillance Vaccination Legal costs Welfare depopulation	Disease control compensation	

Industry			Loss of export market Abattoir losses Loss of animals culled for welfare reasons Withholding Secondary C&D

*IPs, infected premises; DCs, dangerous contacts; C&D, cleaning and disinfection*.

Briefly, the estimate of the direct cost of a given outbreak was directly calculated from outputs of the epidemiological model. In particular, the estimate of the direct cost depends on (i) the numbers of cattle, sheep, and pigs culled for disease control purposes, (ii) the number of premises defined as IPs and depopulated, (iii) the number of premises defined as DCs and depopulated, (iv) the duration (in days) of the outbreak, (v) the total number of doses used during the vaccination campaign (if implemented), as well as (vi) the numbers of farms and animals that have been vaccinated. All epidemiological outputs were then allocated to relevant specific cost elements and directly transformed into economic values based on current relevant international (i.e., from the World Organisation for Animal Health, OIE) and local legislations, control procedures, and guidelines when facing FMD outbreaks. In particular, international trade restrictions were assumed to last for 3 months following the last case in the absence of vaccination, and 6 months when vaccination is used, in line with current EU policy. Pricing information used to estimate each specific cost element was sourced from previously published data (19), adapted or updated where necessary. Discussions with policy makers in Scotland and UK governments and state veterinary organizations were undertaken in 2012 to validate assumptions and ensure that any major changes in policy or strategy were reflected. Table S1 in Supplementary Material provides further details on the specific cost elements considered in the model and the assumptions related to each element.

## 3 Results

**Figure 1:**
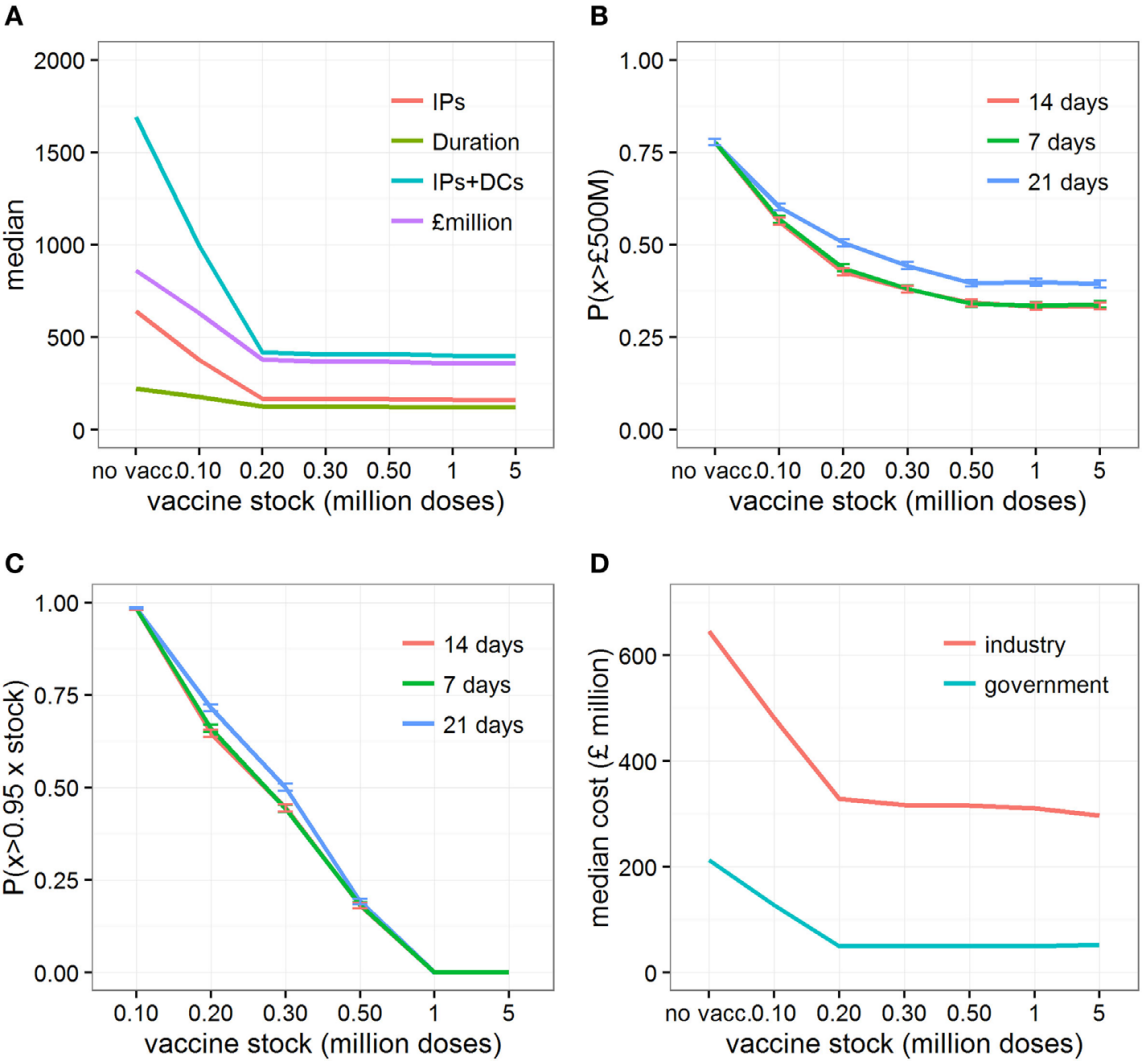
Changes in epidemic and economic outcomes when vaccination is implemented, or not, under conditions conducive to large outbreaks. Changes in **(A)** the median estimates of various epidemic outcomes under various vaccine stocks; **(B)** the probability of the direct cost exceeding *£*500million, P(*x* > *£*500M), depending on days between detection and vaccination; **(C)** the probability of using more than 95% of the initial vaccine stock; and **(D)** the median direct economic costs incurred by each sector when vaccination is not implemented, or implemented 14 days after detection assuming an initial vaccine stock that varies between 100,000 and 5 million doses. Epidemic outcomes shown in **(A)** are number of infected premises (“IPs”), duration of the outbreaks in days (“duration”), number of infected premises and premises identified as dangerous contacts (“IPs + DCs”), and the total direct costs of the outbreak in *£*millions (“cost”). Shown in panels **(B)** and **(C)** are changes in P(*x* > *£*500M) and P(*x* > 0.95xstock) when vaccination is implemented for 7, 14, and 21 days after the detection of the index cases, respectively.

**Figure 2:**
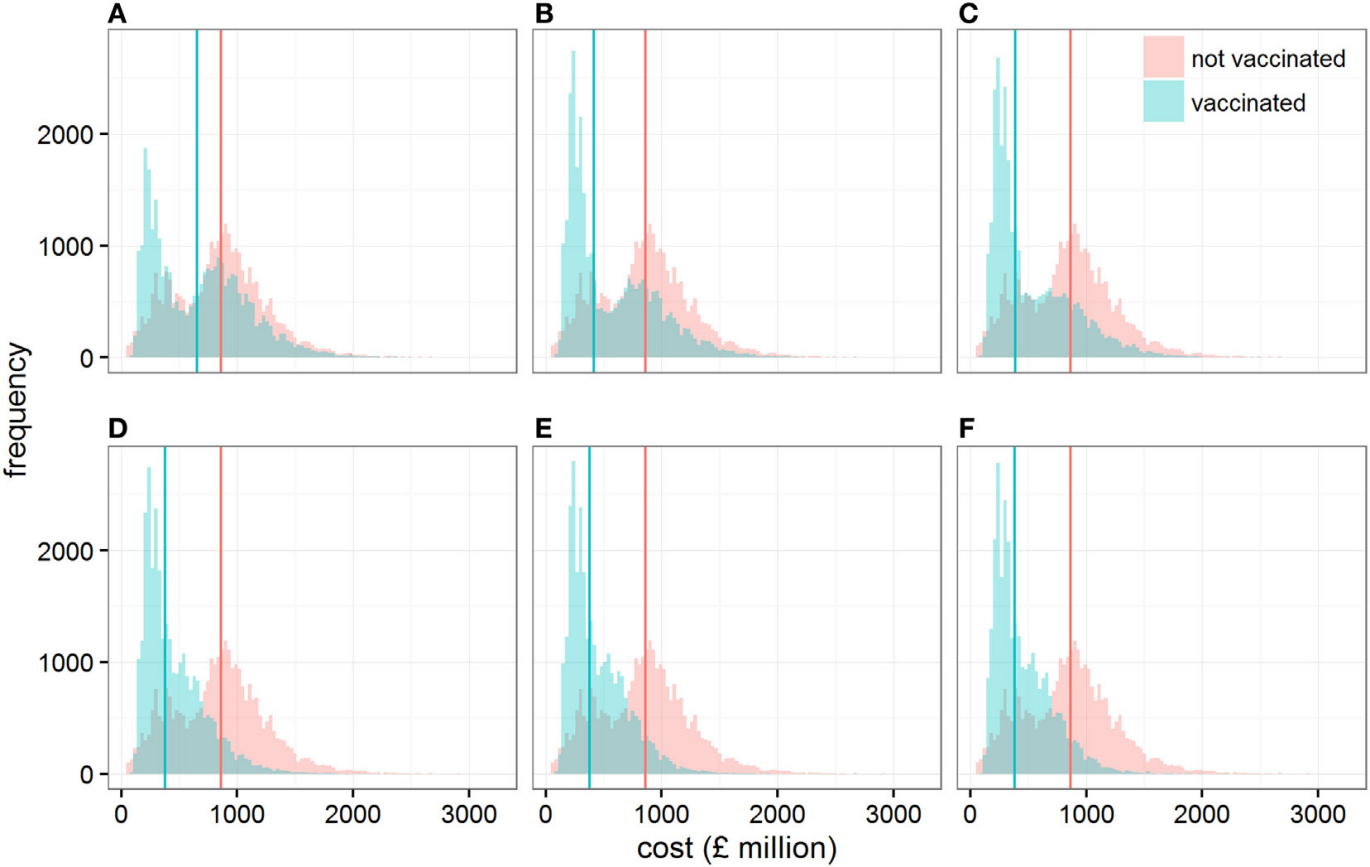
Distribution of the direct economic cost (in *£* million) when cattle are vaccinated or not vaccinated, assuming conditions conducive to large outbreaks. Each panel shows these distributions when the size of the vaccine stock at the start of the epidemic is **(A)** 0.1, **(B)** 0.2, **(C)** 0.3, **(D)** 0.5, **(E)** 1, and **(F)** 5 million doses. Solid vertical lines represent the median direct economic costs in each scenario.

The model results indicated that, for scenarios with conditions conducive to large outbreaks, and in the situation where the initial vaccine supply is limited and restocking is not available, all vaccination strategies were found to perform better than the strategy where only IP/DC is implemented (Figures 1 and 2; Figures S1–S3 in Supplementary Material). However, a stock of 200,000 doses (i.e., 12% of all cattle in Scotland) would be sufficient to maximize the relative benefits of vaccination, both from an epidemiological and an economic standpoint (Figure 1A). Under such situation, completing a vaccination-to-live strategy alongside IP/DC culling would result in a median of 291,049 head of livestock (i.e., cattle, sheep, and pigs) culled (95% range 32,065–1.69 million) at a cost of *£*417 million (95% range *£*155–*£*1,455 million), affecting 490 farms (95% range 84–1,941) and lasting for 139 days (95% range 46–354 days). In comparison, implementing IP/DC alone would result in a median of 1.24 million head of livestock culled (95% range 78,500–2.02 million) at a cost of *£*862 million (95% range *£*169 million–*£*1,701 million) and a median outbreak duration of 221 days (95% range 68–391 days).

At the same time, varying the size of the initial stock of vaccine impacts on the variability associated with the cost of controlling outbreaks (Figure 2). In particular, controlling epidemics through a vaccination strategy with a stock of 200,000 (quartiles coefficient of dispersion QCD = 1.52) to 300,000 doses (QCD = 1.31) generates a wider variation in its economic cost than if vaccination is not implemented (QCD = 0.64). In other words, while the application of vaccination would be beneficial relative to no vaccination on average, we would have less certainty in the outcome. This result may be a source of concern; however, it is due to the vaccination strategy’s ability to progressively reduce the chance of outbreaks requiring large numbers of animals to be culled for disease control (Figure S3 in Supplementary Material).

The probability of direct costs exceeding £500 million, P(*x* > £500M), is reduced when vaccination is used, and steadily reduces further as the size of initial vaccine stocks increases, before plateauing when initial vaccine stocks exceed 500,000 doses (i.e., 30% of all cattle in Scotland), regardless of delays (from 7 to 21 days) in implementing the vaccine-to-live strategy in the field (Figure 1B). Looking at the probability that at least 95% of the initial vaccine stock is used to control the epidemics (Figure 1C), it is apparent that increasing the initial stock of vaccine would potentially leave large volumes of unused vaccine, even in the studied scenarios with conditions conducive to large outbreaks or when delays (from 7 to 21 days) occur in implementing vaccination in the field. For example, 95% of the vaccine stock is used 65% of the time with an initial vaccine stock of 200,000 doses. In contrast, when the stock exceeds 1 million doses (i.e., covering 60% of all cattle in Scotland), 95% of the vaccine stock is used 0% of the time.

**Figure 3:**
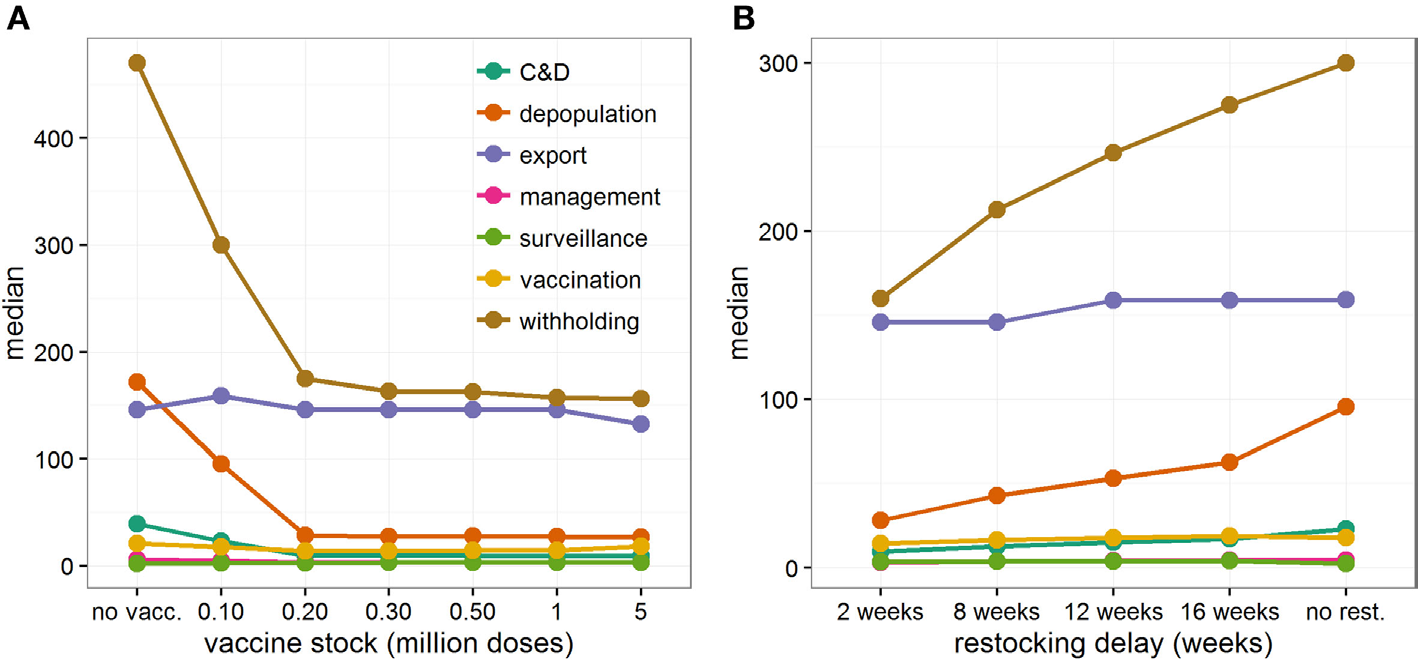
Contribution of each specific cost elements to the total direct economic cost when controlling large FMD outbreaks in Scotland. Median cost estimates (in *£* million) of each specific cost element for **(A)** increasing initial vaccine stocks from 100,000 to 5 million doses, and **(B)** increasing delays in restocking vaccines, from 2 to 16 weeks, when initial vaccine stock was limited to 100,000 doses. Here, vaccination was implemented 14 days after detection of FMD in Scotland. If new stocks of vaccine doses have been ordered **(B)**, restocking demand has been triggered when less than 10% of the initial vaccine stock remains. Considered cost elements are those related to (i) the cleaning and disinfection (C&D) of depopulated farms (including preliminary and secondary C&D), (ii) the depopulation of farms (including compensation and legal costs), (iii) the loss of export trade, (iv) managing disease control activities, (v) the implementation of surveillance activities during and post outbreak, (vi) the implementation of the vaccination-to-live strategy and the reduction in value of vaccinated animals, (vii) the implementation of a national movement ban (including the loss of trade and the reduction in value of withheld animals, losses due to the reduction of throughputs in Scottish abattoirs, and the worsening of animal welfare standard).

Although the cost borne by the industry is six times greater (median: 6.02, 95% range: 2.95–9.90) than the cost incurred by the government in all vaccination scenarios, increasing the size of the vaccine stock at the start of the epidemic would be beneficial for both industry and government (Figure 1D). Figure 3A and Figure S4 in Supplementary Material illustrate the distribution of economic value, and relative contribution to the total estimates, of each specific cost element under different scenarios of initial vaccine stocks. In the situation where initial stock is 200,000 doses, the loss of export market, national movement ban (and its effects on reduced value of animals and increased welfare losses), and livestock culled due to disease control dominate the direct costs of a widespread FMD outbreak in Scotland (Figure 3A), accounting for 38% (95% range: 17–70%), 45% (95% range: 18–68%), and 9% (95% range: 3–21%) of the total cost, respectively (Figure S4 in Supplementary Material). For comparison, the contribution of the loss of export market, movement ban, and livestock culled in overall direct cost of a widespread FMD outbreak in Scotland are 17% (95% range: 12–47%), 56% (95% range: 34–70%), and 17% (95% range: 7–27%), respectively, when implementing IP/DC alone. However, increasing the initial vaccine stock size decreases the costs associated with culling and with movement restrictions (Figure 3A; Figure S4A in Supplementary Material), notably due to reduced duration and number of IPs (Figures S1 and S2 in Supplementary Material). In contrast, increasing the initial vaccine stock size does not impact significantly on the losses of the export market (Figure 3A; Figure S4A in Supplementary Material). However, it increases the importance of the loss of the export market in the total direct costs of the outbreak, accounting on average for nearly half of the total direct cost (Figure S4B in Supplementary Material).

**Figure 4:**
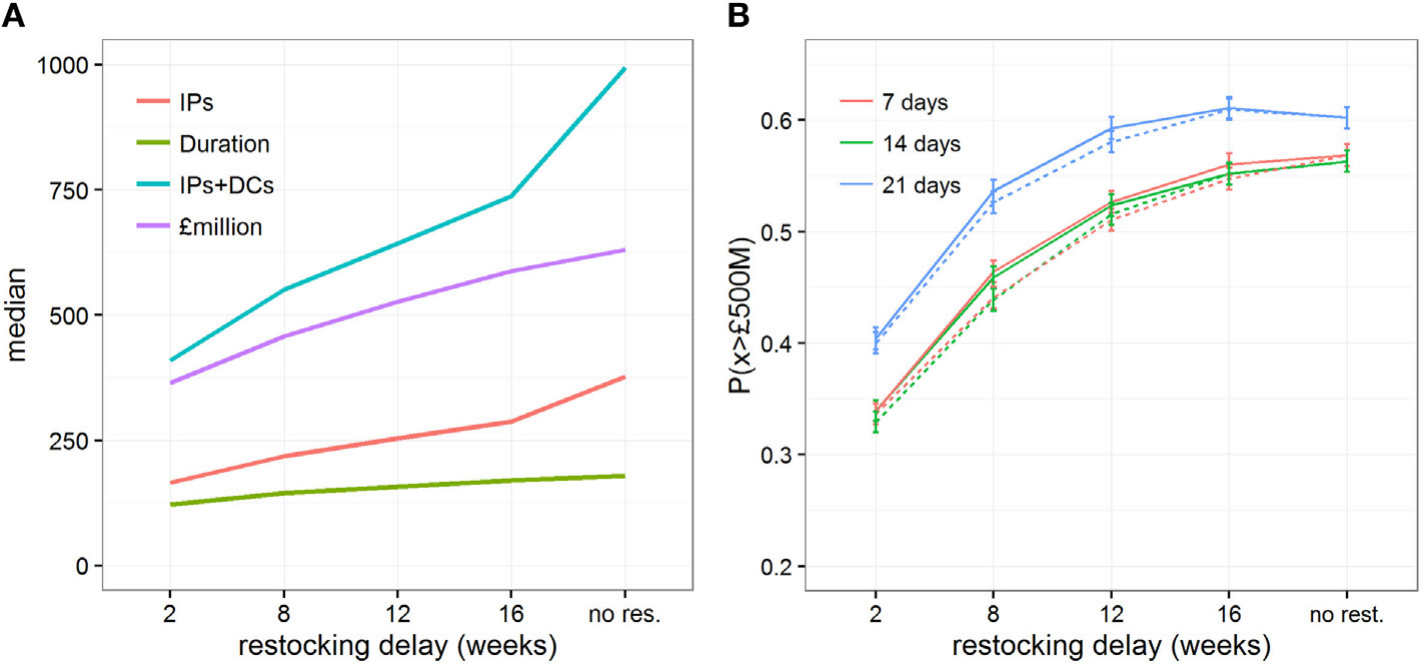
Impact of restocking delays on the epidemic and economic outcomes when implementing a vaccination strategy under conditions conducive to large outbreaks. Changes in **(A)** the median estimates of various epidemic outcomes, and **(B)** the probability of the direct cost exceeding *£*500million, P(*x* > *£*500M), are shown for scenarios which either do not allow a restocking strategy or allow a restocking strategy with increasing delays, from 2 to 16 weeks. Here, initial vaccine stock was limited to 100,000 doses. Solid and dashed lines represent the changes in outcomes when restocking demand is triggered when less than 10% and 50% of the vaccine stock remains, respectively.

When investigating the impact of different lengths of restocking delays, we considered that only a relatively small vaccine stock of 100,000 doses (i.e., covering 6% of all cattle in Scotland) would be initially available to control the outbreak. Figure 4 shows the epidemiological and economic consequences when increasing the length of time required to receive new vaccine stocks, and highlights that large restocking delays are of particular importance. In particular, delays of >2 weeks rapidly increase the size and duration of the outbreaks (Figure 4A; Figures S5 and S6 in Supplementary Material) and increase the direct costs of control from *£*365 million (95% range: *£*151–*£*1,051 million) to *£*588 million (95% range: *£*151–*£*1,205 million, Figure 4A; Figure S7 in Supplementary Material). Looking at the risk of outbreaks costing over *£*500 million (Figure 4B), delays in restocking vaccine doses from 2 to 16 weeks substantially increased P(*x* > £500M) from 0.329 (95% C.I. 0.320–0.338) to 0.552 (95% C.I. 0.542–0.562). This general trend is not disproportionately affected by delays in the decision to vaccinate, though a late decision to vaccinate would ultimately further increase the risk of expensive outbreaks (Figure 4B). It is, however, worth noting that in situations in which the vaccination strategy decision is taken late (i.e., 21 days after detection) with a suboptimal initial vaccine stock, the risk of expensive outbreaks when large (i.e., 12 weeks) restocking delays occur (P(*x* > *£*500M) = 0.593, 95% C.I. 0.583–0.603) would be the same as if no restocking occurred (P(*x* > £500M) = 0.602, 95% C.I. 0.592–0.612), while the median direct cost would be £30.6 million less (i.e., a saving of only 4.4%).

Restocking delays are particularly felt by the government, nearly doubling its average expenses from *£*50.4 to *£*96.6 million. This increase is due to the government facing higher disease control costs and greater demands for welfare depopulation (Figure S8 in Supplementary Material). However, the industry still bear most of the costs, which increase by 45% if restocking delays increase from 2 to 16 weeks. These increases in costs result from the national movement ban being enforced for a longer period of time, causing large welfare losses of animals, and high withholding costs incurred by individual farmers (Figure 3B; Figure S8A in Supplementary Material). By contrast, the increase in loss from reduced exports is relatively small (Figure 3B; Figure S8A in Supplementary Material) and, as a consequence, its contribution to the total direct cost progressively decreases (Figure S8B in Supplementary Material).

**Figure 5:**
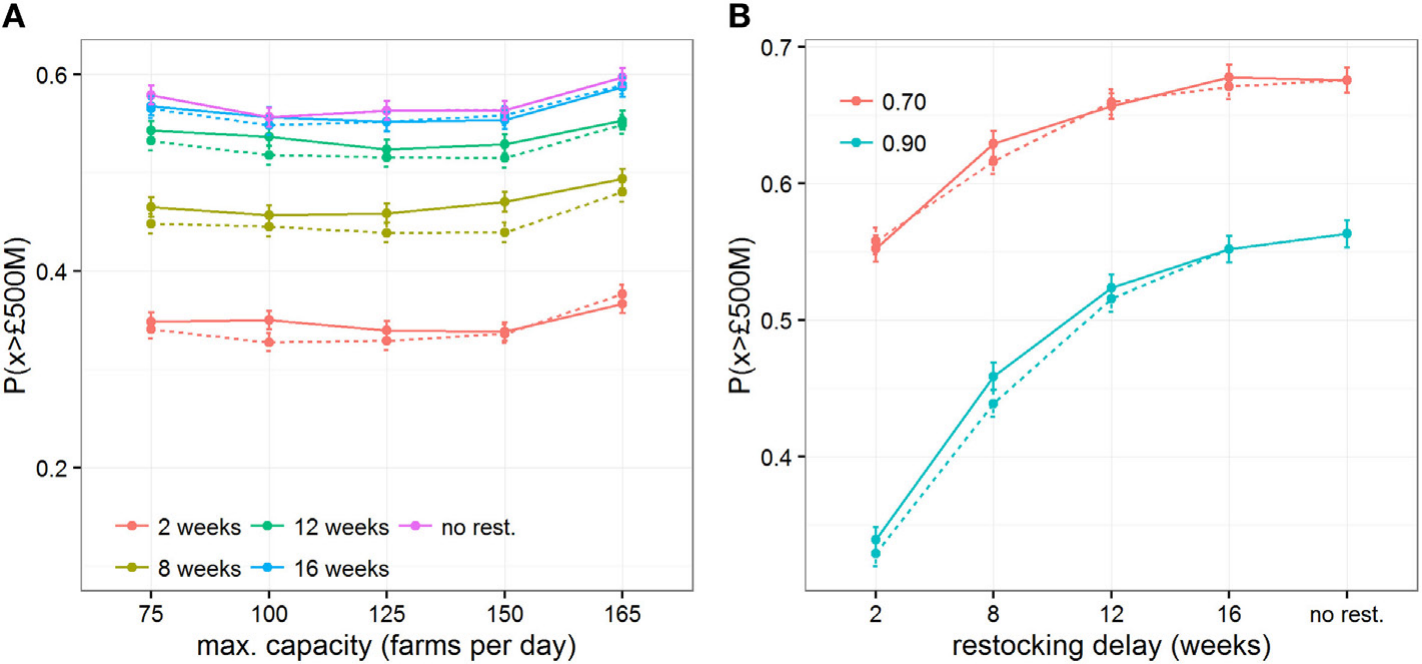
Impact of restocking delays on economic outcomes when implementing imperfect vaccination strategies and under conditions conducive to large outbreaks. Changes of the probability of the direct cost exceeding *£*500million, P(*x* > *£*500M), are shown for varying **(A)** the maximum daily capacity of vaccination teams to vaccinate farms and **(B)** the vaccine efficacy in generating an immune response to cattle. Solid and dashed lines represent the changes in outcomes when restocking demand is triggered when less than 10% and 50% of the vaccine stock remains, respectively.

Finally, we investigated the impact of various operational constraints that may affect the economic outcome of a vaccination strategy with increasing restocking delays: the threshold at which new vaccine stock is ordered, the efficacy of the vaccine, and the maximum daily capacity (in number of vaccinated farms) of vaccination teams. Varying the threshold at which new vaccine stock is ordered from 10 to 50% remaining of the initial stock shows little impact on the risk of very costly outbreaks P(*x* > *£*500M) (Figures 4B and 5) or on epidemiological outcomes (Figures S5 and S6 in Supplementary Material). These results indicate that the point at which new stock is ordered has less impact than the delays in restocking, presumably because the number of days saved by ordering earlier would be minimal in comparison to the length of time taken to restock. Similarly, varying the maximum number of farms vaccinated per day from 75 to 165 does not mitigate the impact of restocking delays (Figure 5A). In contrast, quick restocking may offset, at least partly, economic losses due to poor vaccine efficacy. Indeed, for a vaccine with 70% efficacy, restocking within 2 weeks would reduce P(*x* > £500M) to a similar level as when using a vaccine with 90% efficacy, but with restocking delays exceeding 12 weeks (Figure 5B).

## 4 Discussion

Predictive models have been extensively used worldwide to assess the likely effectiveness of possible vaccination measures in the event of an FMD outbreak (3, 6, 8, 11, 17, 20–25). With such models, several operational aspects of an FMD vaccination strategy are now better defined. For instance, the choice of implementing a vaccination strategy ultimately depends upon the early infection profile and the perceived likelihood of a large-scale epidemic (3, 26). However, the use of vaccination during an FMD outbreak remains limited by uncertainties regarding the availability of vaccine stocks and their dynamics, particularly with regards to their impact on the final economic cost for government versus industry. Here, we have explored how capacity constraints may affect the cost-efficiency of a vaccination-to-live strategy during an FMD outbreak in Scotland.

Our results show that, for scenarios with conditions conducive to large outbreaks, all vaccination strategies were economically beneficial compared to a strategy where only culling of infected premises and dangerous contacts was implemented. These results not only reaffirm findings from studies in Europe and elsewhere that vaccinating animals to support culling strategies can be beneficial epidemiologically (3, 23, 27), but also indicate that these strategies can be economically beneficial when controlling widespread epidemics.

Even when vaccination is used, there is still a risk of very costly outbreaks in Scotland (i.e., >£500 million). This risk is most reduced when an initial stock of at least 500,000 doses, sufficient to vaccinate 30% of all cattle in Scotland, is available as soon as FMD is detected. The costs saved when 500,000 doses are available are mostly due to reduced depopulation activities and shorter duration of movement restrictions (Figure 3; Figure S4 in Supplementary Material). On the other hand, costs associated with the loss of the export market remain unaffected, becoming the most substantial relative cost (>45%; Figure S4 in Supplementary Material) as other costs reduce. Even though the loss of the export market is important, it is not critical enough to affect the relative benefits of using vaccination to control large epidemics in Scotland. This is mostly because commodities subject to international restrictions during FMD outbreaks represent a relatively small proportion of Scotland’s GDP compared to other countries. In contrast, for export-focused countries, such as Denmark, vaccinate-to-live strategies are not cost-effective (28). This is particularly due to current regulations restricting exports for 6 months before regaining free status when vaccination is used, rather than 3 months when only “stamping out” is used. The necessity of these restrictions has been questioned (29); clearly any changes to this policy could have significant impacts on the cost-effectiveness of vaccination strategies.

While a risk-averse policymaker might focus on minimizing the risk of a very costly outbreak (by stocking at least 500,000 doses), we have shown that a stock of 200,000 doses (i.e., covering 12% of all cattle in Scotland) would be sufficient to maximize the relative benefits of vaccination, both from an epidemiological and an economic standpoint, and to minimize losses due to vaccine stock wastage (Figure 1). In the case that sufficient vaccine is not immediately available, restocking is an option to optimize vaccination benefits. However, constraints in sourcing and shipping new stocks may create delays. Here, we have shown that delays in restocking would increase the cost and duration of an outbreak. Notably, delays of more than 56 days not only increase the size, duration, and direct economic cost of the FMD outbreak at hand, but also increase the risk of the direct economic costs exceeding *£*500 million. The effects of vaccine restocking delays on outbreak duration drive a relative increase in costs associated with movement restrictions, while the increased number of IPs increases the costs associated with culling for disease control. This suggests that if only small initial stocks are available, vaccination should still be implemented, but the availability and ability to draw from existing FMD VACCINE stocks, whether in Scotland, in the rest of UK or overseas, must be considered to ensure delays are minimized.

In reality, the batch size and cost of vaccine purchases would likely be subject to individual negotiation, availability of appropriate antigen strains, and concurrent FMD vaccine requirements in other countries. Our results highlight that priorities regarding vaccine access are (i) the number of vaccine doses available at the start of an outbreak and (ii) the speed of restocking. In addition, the interplay with vaccine efficacy and delays in the field implementation of the vaccination strategy is important. Delays in vaccine restocking become particularly important when facing an outbreak of a serotype where low vaccine efficacy is a concern. Therefore, vaccine availability and efficacy should be considered together when deciding whether vaccination should be implemented.

In a previous study, we showed that delays in implementing vaccination reduce its epidemiological benefit (3). In this study, we found that implementing vaccination at 21 days compared to 7 or 14 days increased the risk of very costly outbreaks, regardless of the size of vaccine stock available. Given the time needed to source vaccine and initiate implementation, some delays are difficult to avoid. Vaccination is usually beneficial only in large outbreaks (3). This study considered only scenarios where conditions conducive to large outbreaks, hence vaccination was likely to be beneficial. In reality, decisions about whether to vaccinate or not have to be made based on only the initial epidemiological picture in order to minimize the delay. The first fortnight incidence (30) and first 14 days of spatial spread (24) have been described as indicators of the likely size and duration of an epidemic, which can be used to make a decision about whether to implement vaccination. Our results show that there is little difference between implementing vaccination at 7 days compared to 14 days, regardless of the vaccine stock available, confirming that taking 14 days to assess the epidemiological picture before making a decision about vaccination would not significantly affect the benefits.

In the scenarios we looked at, which predisposed for large outbreaks, the livestock industry always bore more than double the costs of the government. However, this relative cost burden to industry increased to four times that of government when vaccination was used. In previous FMD outbreaks, the Scottish/UK Governments were eligible for rebates from EU for some aspects of disease control and compensation costs (19). Given the uncertainty over Scotland’s future relationship with EU, this rebate was excluded from our analyses. If any rebate was available, however, this would have reduced costs borne by the Scottish government alone. Our findings that the industry would bear a substantial majority of costs are in contrast to some previous findings, that in a large outbreak in GB, over half the costs sit with government (19), but in line with the relative distributions described by other authors (31, 32).

In our model, the costs to the industry are particularly driven by the effects of movement restrictions and the impact of export bans. Movement licenses are issued during an outbreak to allow specific movements to occur, particularly movements to slaughter, to limit the negative economic and animal welfare consequences. For simplicity, we assumed that all animals intended for slaughter were kept for 30 days before licenses were issued or until the outbreak ended, whichever occurred first. We made such an assumption in line with current policy, but in reality movement restrictions and licensed moves are more fluid and responsive as they depend on both the epidemiological and political context at the time. Notably, licenses can be issued in a phased approach, with for example movement of animals to slaughter licensed for specific geographical areas at least 8 days after the most recent IP (33). However, when looking in more detail at the number of animals subject to movement restrictions, less than 20% were intended for slaughter (18.7%, 95% range 10.4–38.7%) and, therefore, eligible to be moved under license to slaughter. Hence, although we may have overestimated losses due to movement restrictions, such a bias should be limited and should not significantly affect our overall results.

In our economic model, we assumed that animals and products that could not be exported would be slaughtered and consumed within the domestic market, incurring a loss in value. This contributes to the larger proportion of costs borne by industry when vaccination is used, since vaccination incurs a 6-month trade restriction. It is difficult to predict how markets would behave in an outbreak situation where vaccination is used. An assessment of the effect on markets and impacts on related direct costs (such as tourism or other rural industries) are, therefore, important, but beyond the remit of this study. Other authors have assumed that trade with other EU countries could continue from non-affected regions, if regional approaches were permitted (28, 31). In the model, we ignored the potential impact of applying the principle of regionalization (as defined by EU Council Directive 2003/85/EC) on within-EU trade when emergency vaccination is conducted, meaning that all trade with other EU members was assumed to not be possible. However, if some trade were possible, the cost of trade restrictions would be reduced, further increasing the economic benefit of implementing emergency vaccination to control FMD (28).

In this study, we assumed that an initial supply of vaccine would be available shortly after FMD is declared in Scotland, regardless of the strain and serotype of the virus involved in the outbreak. Quick access to vaccine can be achieved by calling upon national or international bank(s) of fully formulated FMD vaccines and/or FMD antigen (34, 35). The UK decision to leave the EU (known as “Brexit”) has introduced uncertainty regarding the ability of the UK to access European and international vaccine banks (36). Opportunity costs associated with formulating, maintaining, or purchasing vaccine stock were not included in our model, but could be significant (37). Whether these costs would offset or not the economic benefit of an emergency vaccinate-to-live strategy is unclear and, therefore, needs to be considered in the future.

Our results have implications for making robust decisions on how best to control FMD in Scotland. When comparing potential FMD control strategies, metrics used to assess outcomes are important. Different metrics give different optimal strategies (38) and in reality reflect the priorities of different stakeholders (31). Here, we chose to estimate the full economic cost of activities when controlling a FMD outbreak. While calculating all direct costs is time-consuming, it is more likely to reflect the reality of the range and interplay of impacts than simply using epidemiological outcomes (such as the number of IPs, or duration of the outbreak (3)), or simplified indicator costs (25, 31, 38). This is particularly relevant when it comes to incorporating factors, such as trade bans. For example, a recent study from Denmark highlighted that emergency vaccination was never cost-effective due to impacts on the substantial Danish export market, despite being epidemiologically effective (28). In addition, measures assessing cost-effectiveness are often required for policy makers to make decisions; thus, it is helpful to present a range of outcomes (epidemiological and economic) to demonstrate the issue’s complexity to policy makers.

In conclusion, our findings confirm that an emergency vaccination-to-live strategy, in addition to the conventional stamping out strategy, is economically beneficial in situations conducive to large outbreaks in Scotland. However, the size of the initial vaccine stock available at the start of the outbreak, and the interplay with other factors, such as vaccine efficacy and delays in implementing or restocking vaccination, should be considered in making decisions about optimal control strategies for FMD outbreaks.

## Author Contributions

TP, HA, and KR designed the study, and drafted the manuscript. TP and HA developed the direct cost estimator. TP carried out the modeling. All authors gave final approval for publication.

## Conflict of Interest Statement

The authors declare that the research was conducted in the absence of any commercial or financial relationships that could be construed as a potential conflict of interest.
