# Supplementary material for: Assessing the Economic Impact of Vaccine Availability When Controlling Foot and Mouth Disease Outbreaks
Source: Front Vet Sci. 2018 Mar 13;5:47. doi: 10.3389/fvets.2018.00047 (PMC5859371; doi:10.3389/fvets.2018.00047)
Supplement: Supplementary file 1 [file Presentation_1.PDF]

frontiers

Supplementary Material: Assessing the economic impact of vaccine availability when controlling foot and mouth disease outbreaks

SUPPLEMENTARY TABLES

Table S1: Details of all components involved in the foot and mouth direct cost calculator.

**Cost category**	**Costs**	**Details**	**Reference**
Costs to government	Management	A National Disease Control Centre and a Local Disease Control Centre are set up to coordinate disease control activities. These costs are a function of the duration of the epidemic and based on the labour costs previously previously published.	Risk Solutions (2005); DEFRA (2011)
	Identifying IPs and DCs	The cost of identifying infected premises (IPs) and tracing dangerous contacts (DCs) relates to the labour cost of visiting and tracing IPs and DCs, and required diagnostic tests.	Risk Solutions (2005)
	Depopulation	This includes costs associated with culling and disposal, plus other visits such as valuation, machinery hire and carcass transport and disposal. Labour costs were related to either the number of animals or the number of farms depopulated, depending on the specific activity. This includes "headage" payments (additional labour payments based on the number of animals culled). The hiring of plant and machinery needed for culling was calculated according to the total number of farms that were depopulated. Carcass disposal and transport were related to the weight of dead animals (as defined by the average weight of carcasses) and the daily capacities of incineration and rendering facilities available, as well as the average carcass load capacity for a truck.	Risk Solutions (2005); Expert opinion
	Preliminary cleaning and disinfection (C&D)	The costs of cleaning and disinfection were calculated using a depopulated premise as the unit of measurement. This includes labour costs associated with C&D and the compensation costs associated with seizure of other materials such as feed. Preliminary C&D costs are covered by the Government.	Risk Solutions (2005); DEFRA (2011)

Continued on next page

Table S1 – continued from previous page

Cost category	Costs	Details	Reference
	Surveillance	Surveillance is required to regain disease freedom. If vaccination is not used, surveillance requirements are for all sheep only premises within the protection zone (PZ) to undergo clinical examination and blood sampling sufficient to detect a 5% prevalence with 95% confidence, and within the surveillance zone (SZ), for a sample of sheep only premises to undergo blood sampling sufficient to detect a infected flock if the flock prevalence is 2% with 95% confidence, and a within flock prevalence of 5% with 95% confidence, using the standard structural protein ELISA. In the model, we could not regionalise the surveillance strategy, so we assumed that all herds in the country were eligible for the surveillance programme. The number of flocks to be sampled was estimated based on the number of sheep flocks remaining after the epidemic and the herd sensitivity of the ELISA test. The number of sheep per tested flock was estimated based on the average number of sheep per flock and the sensitivity of the ELISA test. If vaccination is used, in addition to these requirements, all animals on vaccinated farms must be tested serologically using a serological test for FMD non-structural proteins (NSP) to identify animals that have been infected. As such, the cost of surveillance to prove disease freedom was the sum of the cost of testing the sample of sheep with an ELISA test, and the cost of labour, plus the cost arising from testing all vaccinated cattle farms with a NSP test, if vaccination was used. This is likely to represent an overestimation of surveillance costs, since it does not incorporate zoned surveillance.	Chénard et al. (2003); EU Council Directive 2003/85/EC
	Vaccination	Costs of vaccination comprise the cost of mobilising and demobilising vaccination teams as well as costs relating to keeping vaccination teams on stand-by, regardless of animals being vaccinated or not. While the costs of mobilising and demobilising vaccination teams were fixed by the Agreement Framework, we assume that vaccination teams would remain on stand-by throughout the entire duration of the epidemic at a pre-defined daily cost. The Agreement Framework includes the cost of pre-vaccination visits, but not the cost of the vaccine.<br>Vaccine costs were calculated per dose, based on the values provided for a 500,000 batch. Each vaccine batch purchased (e.g. 100,000 doses) was then assumed to require the total cost, even if all 100,000 doses were not used.	Risk Solutions (2005); Expert opinion
	Legal costs	The costs of legal challenges was calculated using a daily pro-rata legal cost based on costs incurred in the 2001 FMD epidemic.	Risk Solutions (2005)
	Disease control compensation	It was assumed that the government will compensate for animals culled for disease control purpose at the average market price.	QMS (2011); BPEX (2011)

Continued on next page

Table S1 – continued from previous page

Cost category	Costs	Details	Reference
	Welfare depopulation	The government would only consider introducing a Livestock Welfare Disposal Scheme to facilitate culling and disposal of animals as an absolute last resort when all other options have been exhausted, and only if necessary to prevent an unacceptable deterioration in welfare standards. If introduced, a disposal scheme would only apply to animals that cannot be moved under license to a slaughterhouse. No compensation would be offered to farmers for animals culled for welfare issues, but the cost related to the culling itself (i.e. the culling cost, costs of hiring material, transport and disposal costs) would be borne by the central government. We considered the cost of welfare culling supported by the government was a fraction of the depopulation cost due to disease control.<br>For practicality, we assumed that the fraction of the cost of depopulation due to welfare culling depended on both the outbreak duration and the type of disease control measure implemented such that:<br>• given an outbreak lasting less than 31 days, the fraction was zero;<br>• given an outbreak lasting between 31 and 120 days, the fraction would be 0.09 if vaccination is being carried out and 0.04 otherwise;<br>• given an outbreak lasting longer than 120 days, the fraction would be 0.07 if vaccination is being carried out and 0.04 otherwise.<br>The lower limit of 30 days for activating the Livestock Welfare Disposal Scheme was defined as it (1) corresponds to the production cycle for pigs and (2) it represents the minimum length of time for requesting derogation for movements under licence to slaughter. The intermediate limit of 120 days (i.e. 4 months) was set in concordance with the situation in the 2001 epidemics, during which welfare culling typically occurred after 80 days.<br>The rational for different fractions between the situations where vaccination is carried out on the field and that it is not, is based on the assumption that more culling for disease control would be going on leaving less need for welfare culling. Similarly, if the epidemic last for a long period, more animals would be culled and proportional less animals would suffer welfare issues.	
Costs to livestock industry	Withholding costs	We assumed that the overall withholding cost would not result from the extra feed and extra labour used to maintain withheld animals because extra feed costs for one farmer would be balanced against feed savings for another. Instead, the withholding cost was assumed to be the cost resulting from the daily deterioration in quality experienced amongst breeding and stored animals. The number of cattle, pigs and sheep that would be stuck on farm was therefore adjusted to value such deterioration as estimated by Moxey. The total number of animal days stuck, was calculated using the average number of movements per day from farm to farm and from farm to slaughter for the first 30 days (after removal of the number of culled animals), and between farm after the 30 days, with the assumption that movements to slaughter would be licensed after the first 30 days.	Risk Solutions (2005); Moxey (2008)

Continued on next page

Table S1 – continued from previous page

Cost category	Costs	Details	Reference
	Secondary C&D	Although government covers the costs of preliminary C&D, it is assumed that farmers would cover secondary C&D that is required before restocking can be considered. We assumed that the secondary C&D of IPs and DCs would be paid by farmers at a fixed price per premises.	Risk Solutions (2005); DEFRA (2011)
	Loss of value of products from vaccinated animals	It is assumed that vaccination will not impact on the price or saleability of milk in domestic markets since no additional processing is required. There are additional requirements on processing of meat from vaccinated animals to meet EU regulations. Combined with the market impacts of it being unlikely that it would be possible to export meat from vaccinated animals, vaccination has been estimated at reduce carcase value to 10% of normal value. We assumed that the number of vaccinated cattle slaughtered is proportional to the replacement rate per production type. The number of vaccinated cattle head per production type was assumed to be proportional to the national distribution of production type.	Risk Solutions (2005); EU Council Directive 2003/85/EC; UK Statuary Instrument (SI) No. 182 of 2006); UK SI No. 183 of 2006; Expert opinion
	Export ban	Based on EU legislation, a ban on export trade in live animals and animal products would occur. The length of ban depends on whether vaccination was used. If vaccination is used, the ban would be lifted six months after the last infected case or after the last vaccinated animal. In situations where no animals have been vaccinated, the ban remains in place for 3 months after the last infected case. Disaggregated data on export of specific live animals and animal products are not available for Scotland. Therefore, in order to obtain an estimate of Scotland's lost export trade resulting from an export ban, we first calculated the proportion of monthly UK-level export values of each species of live animal and of meat and dairy (i.e., species which were thought to be directly affected by the export ban, i.e., cattle, pigs, and sheep) in the total monthly UK-level value of exports of a relevant broad category ("live animal", "meat and meat preparation" and "dairy and egg"). These monthly proportions were then used to estimate Scotland's monthly species-level export values of live animals, meat, and dairy. Assuming that the export value of given month is a linear product of the daily export value, we then multiply these daily export values by the duration of the international export market closure.<br>It was assumed that animals that would have gone for slaughter for export, instead went to the domestic market (other than those that were culled), valued at the same rate as compensation given.	HMRC (2017, 2011); 2003/85/EC

Continued on next page

Table S1 – continued from previous page

Cost category	Costs	Details	Reference
	Loss of animals culled for welfare reasons	If implemented, no compensation would be offered to farmers for animals culled for welfare issues under the Livestock Welfare Disposal Scheme. Therefore, all costs due to the loss of production and value of animals that were culled as part of this scheme would be borne by farmers. We made the conservative assumption that farmers will lose as much per animal as if it was culled for disease control purpose at the average market price.	QMS (2011); BPEX (2011)
	Losses to others, e.g. abattoirs	With disease control activities, a large volume of animals will be slaughtered for disease control activities. As some of these animals were intended for the sale of meat, their losses would generate a loss of throughput for abattoirs and therefore meat supplies would be replaced by pre-packed imports. Assuming that the volume of throughput affected is proportional to the number of animals culled for disease control, we can then calculate the loss of income to abattoirs and meat processors by multiplying the production volume affected by the outbreak by an assumed gross margin of 15%, estimated from Meat and Livestock Commercial data .The production volume was calculated using average number of movements to slaughter outwith an outbreak, minus animals culled, but including animals that would normally have been exported live.	Risk Solutions (2005)

SUPPLEMENTARY FIGURES

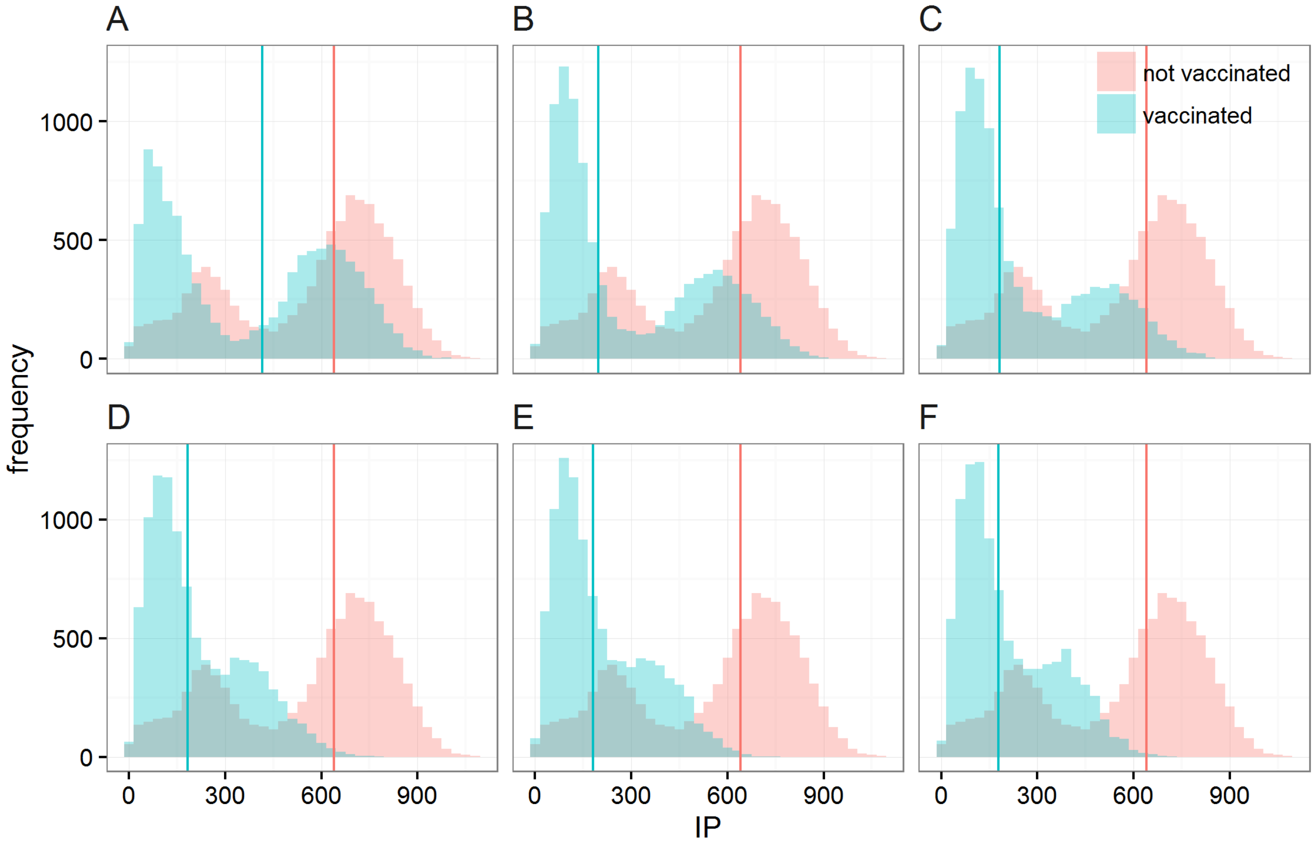

Figure S1: Distribution of the outbreak size when vaccination is implemented or not under conditions conducive to large outbreaks of foot and mouth disease in Scotland. Outbreak size is defined as the total number of infected premises (IP) detected during outbreak investigations. Each panel shows these distributions when the size of the vaccine stock at the start of the epidemic is (A) 0.1, (B) 0.2, (C) 0.3, (D) 0.5, (E) 1 and (F) 5 million doses. Solid vertical lines represents the median direct economic costs in each scenario.

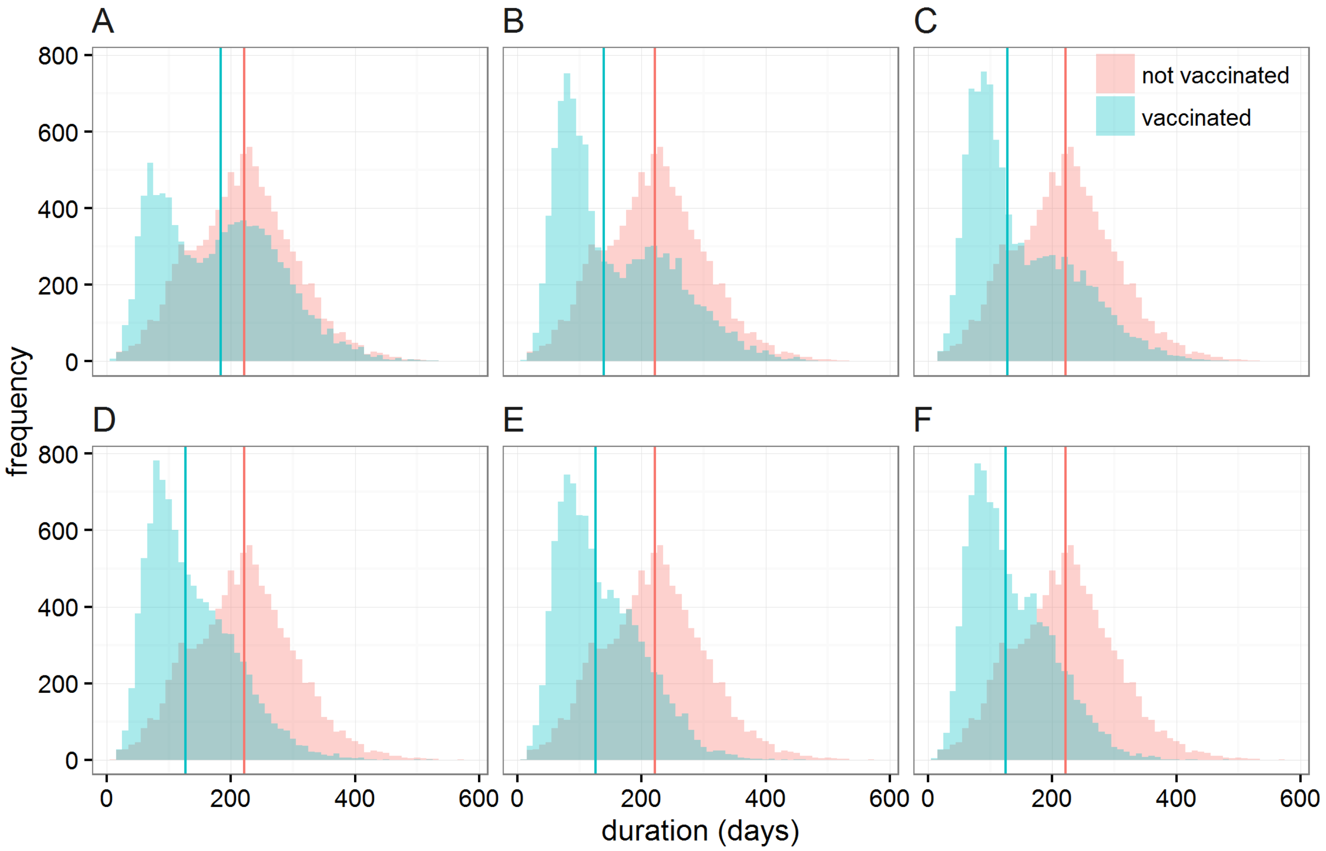

Figure S2: Distribution of the outbreak duration when vaccination is implemented or not under conditions conducive to large outbreaks of foot and mouth disease in Scotland. Outbreak duration is defined as the length of time (in days) between the detection of the disease and the control of the last infected premises. Each panel shows these distributions when the size of the vaccine stock at the start of the epidemic is (A) 0.1, (B) 0.2, (C) 0.3, (D) 0.5, (E) 1 and (F) 5 million doses. Solid vertical lines represents the median direct economic costs in each scenario.

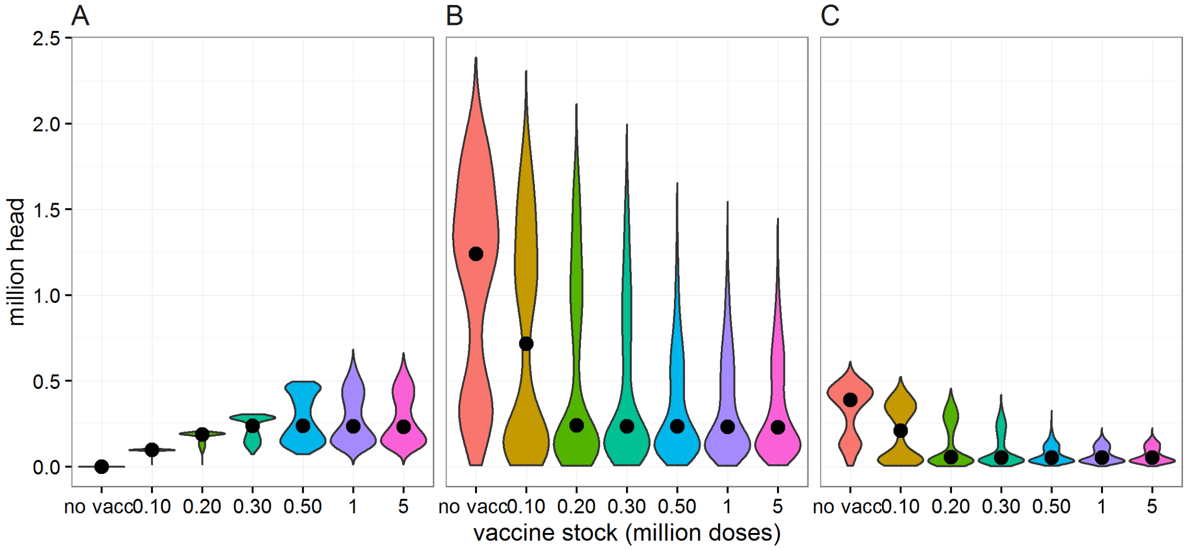

Figure S3: Distribution of animal-level epidemiological outcomes when vaccination is implemented or not under conditions conducive to large outbreaks of foot and mouth disease in Scotland. Violin plot showing the distributions of (A) the number of cattle vaccinated, (B) the total number of animals (cattle, sheep, pigs) culled, and (C) the number of cattle culled when controlling large FMD outbreaks in Scotland and when the size of the vaccine stock at the start of the epidemic varies from 100,000 to 5 million doses. Solid black dots represents the median epidemiological outcome in each scenario.

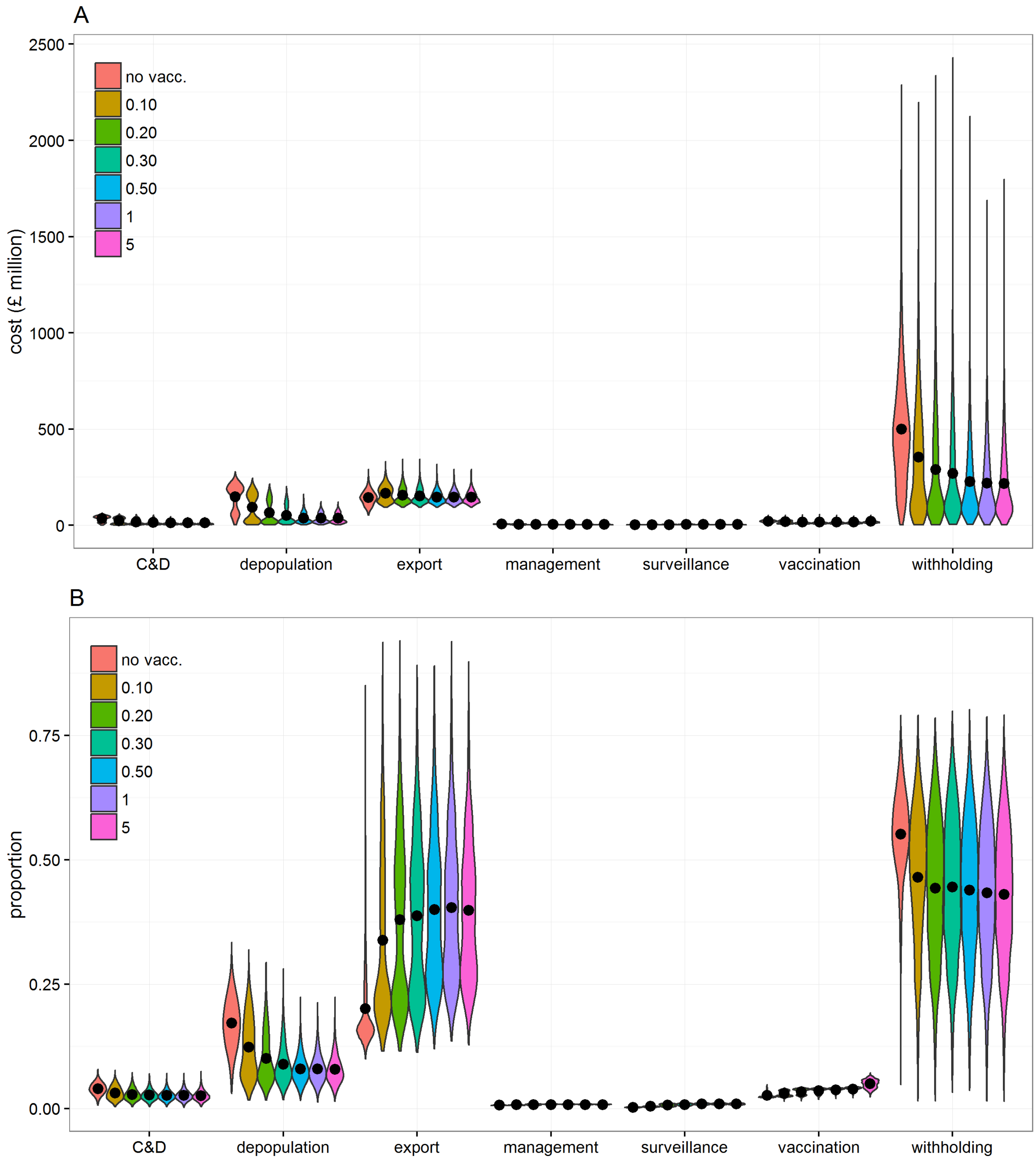

Figure S4: Impact of the size of the initial vaccine stock on the contribution of each specific cost elements to the total direct economic cost when controlling large of foot and mouth disease outbreaks in Scotland. (A) Cost estimates (in million GBP) of each specific cost element. (B) Contribution (as a proportion) of each specific cost element to the total direct economic cost. Here, vaccination was implemented 14 days after detection of FMD in Scotland. Considered cost elements are those related to (*i*) the cleaning and disinfection (C&D) of depopulated farms (including preliminary and secondary C&D), (*ii*) the depopulation of farms (including compensation and legal costs), (*iii*) the loss of export trade, (*iv*) managing disease control activities, (*v*) the implementation of surveillance activities during and post outbreak, (*vi*) the implementation of the vaccination-to-live strategy and the reduction in value of vaccinated animals, (*vii*) the implementation of a national movement ban (including the loss of trade and the reduction in value of withheld animals, losses due to the reduction of throughputs in Scottish abattoirs, and the worsening of animal welfare standard).

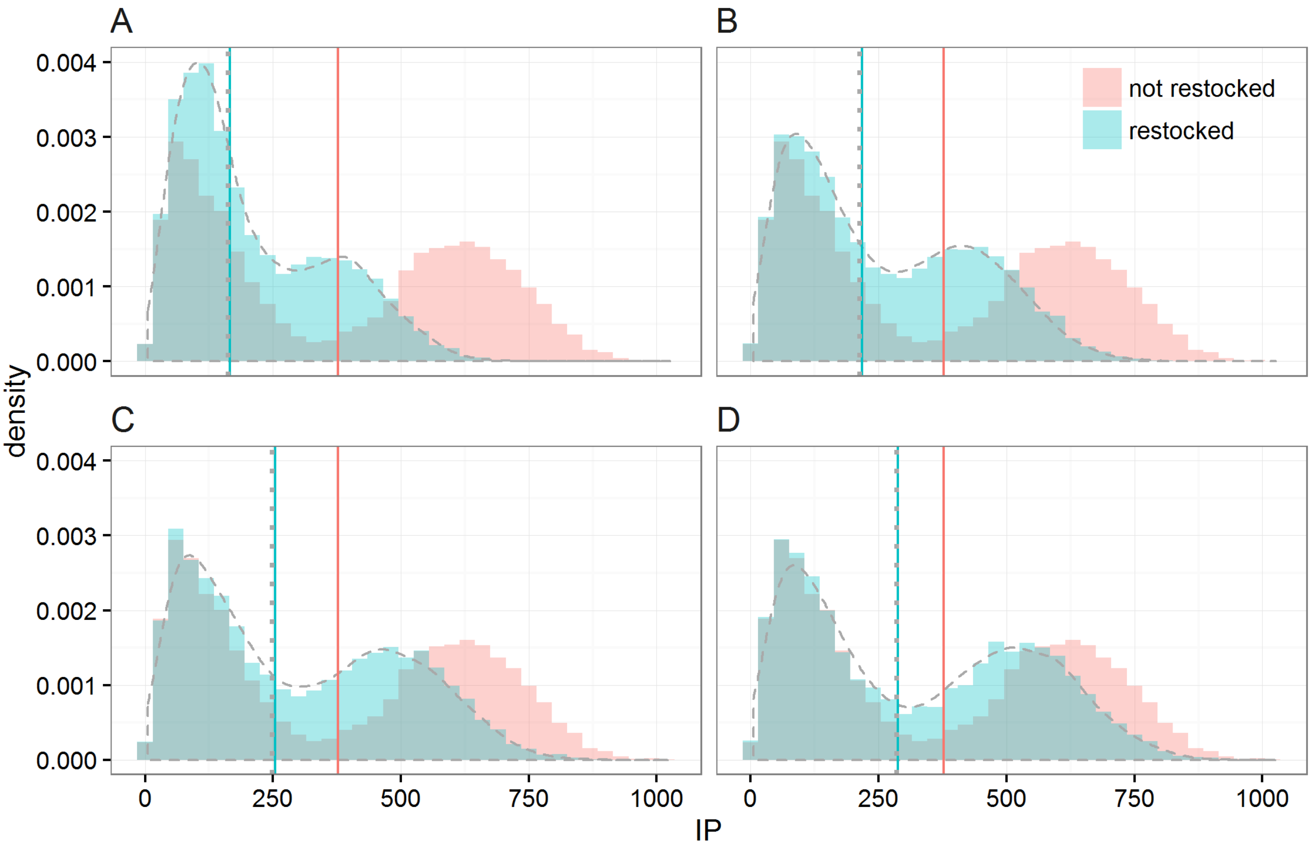

Figure S5: Distribution of the outbreak size comparing when either vaccination was implemented or not and when the initial stock of vaccine is limited to 100,000 doses and restocking demand has been triggered. Outbreak size is defined as the total number of infected premises (IP) detected during outbreak investigations. Each panel shows distributions when a (A) 2-week, (B) 8-week, (C) 12-week, and (D) 16-week delay is required for re-stocking vaccine. Here, restocking demand has been triggered when less than 10% of the vaccine stock remains. Solid vertical lines represents the median epidemic duration in each scenario. For comparison, the distribution of the direct economic cost (grey dashed density curves) and its median (grey dotted vertical lines) when restocking demand is triggered when less than 50% of the vaccine stock remains are also shown.

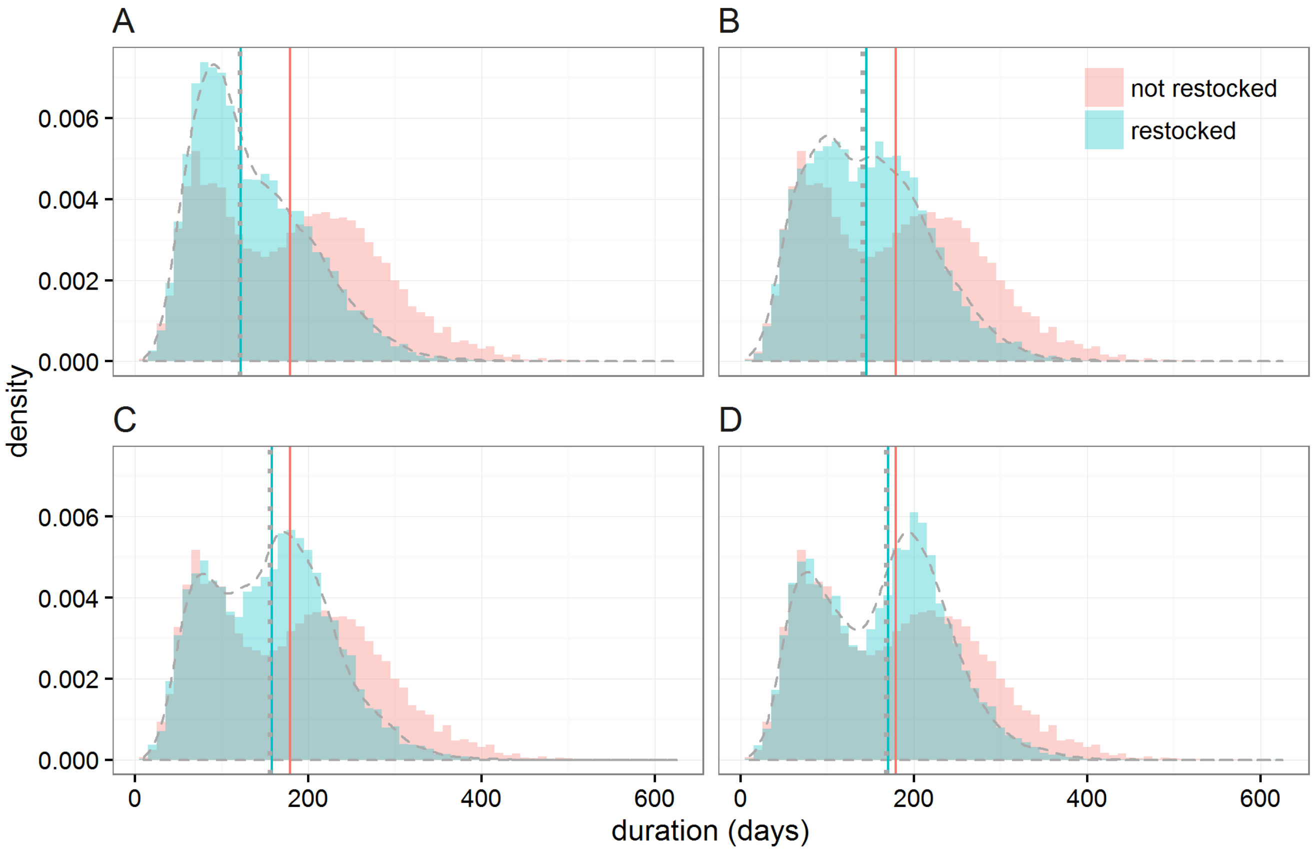

Figure S6: Distribution of the epidemic duration when vaccination was implemented with an initial stock of 100,000 vaccine doses and comparing when restocking demand has been either triggered or not. Outbreak duration is defined as the length of time (in days) between the detection of the disease and the control of the last infected premises. Each panel shows distributions when a (A) 2-week, (B) 8-week, (C) 12-week, and (D) 16-week delay is required for re-stocking vaccine. Here, restocking demand has been triggered when less than 10% of the vaccine stock remains. Solid vertical lines represents the median epidemic duration in each scenario. For comparison, the distribution of the epidemic duration (grey dashed density curves) and its median (grey dotted vertical lines) when restocking demand is triggered when less than 50% of the vaccine stock remains are also shown.

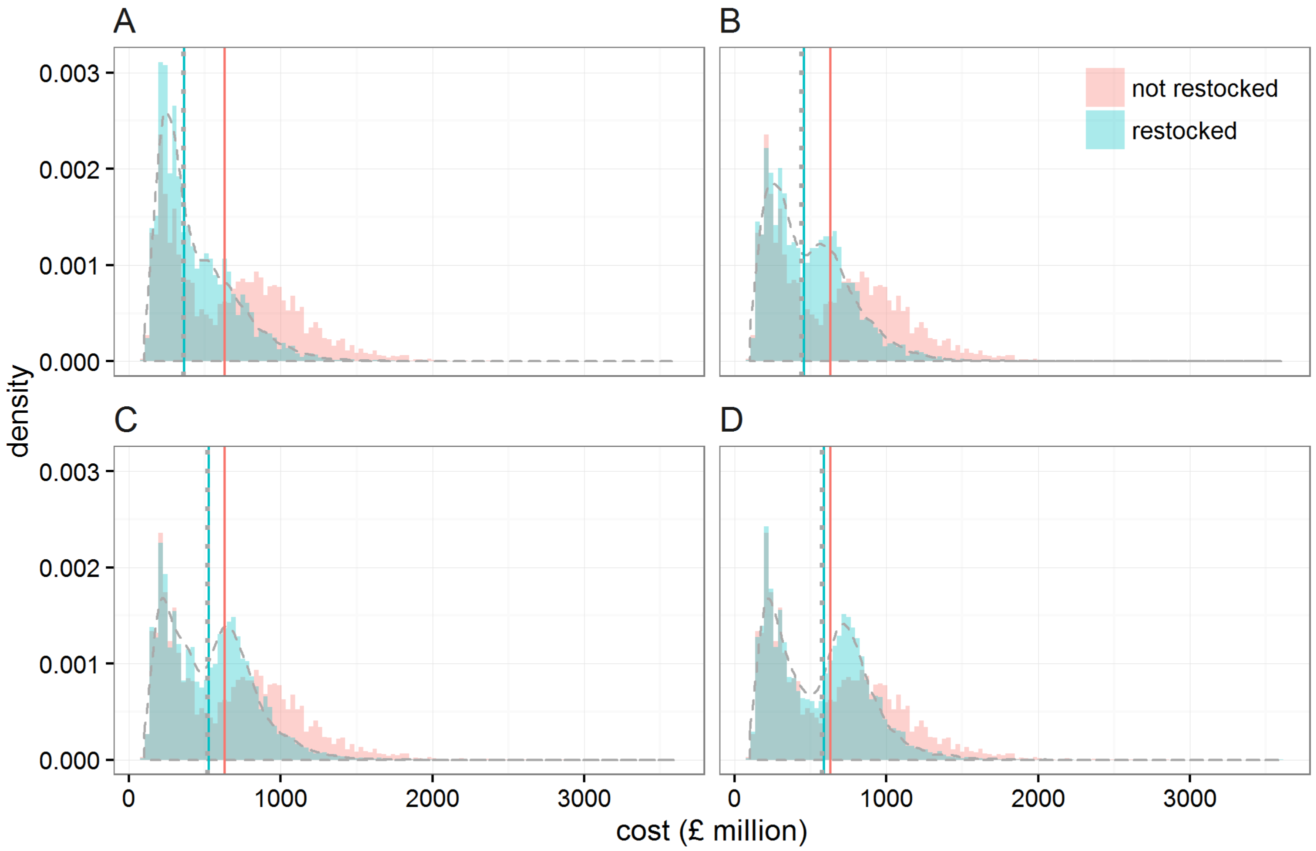

Figure S7: Distribution of the direct economic cost (in million GBP) when vaccination was implemented with an initial stock of 100,000 vaccine doses and comparing when restocking demand has been either triggered or not. Each panel shows distributions when a (A) 2-week, (B) 8-week, (C) 12-week, and (D) 16-week delay is required for re-stocking vaccine. Here, restocking demand has been triggered when less than 10% of the vaccine stock remains. Solid vertical lines represents the median epidemic duration in each scenario. For comparison, the distribution of the epidemic duration (grey dashed density curves) and its median (grey dotted vertical lines) when restocking demand is triggered when less than 50% of the vaccine stock remains are also shown.

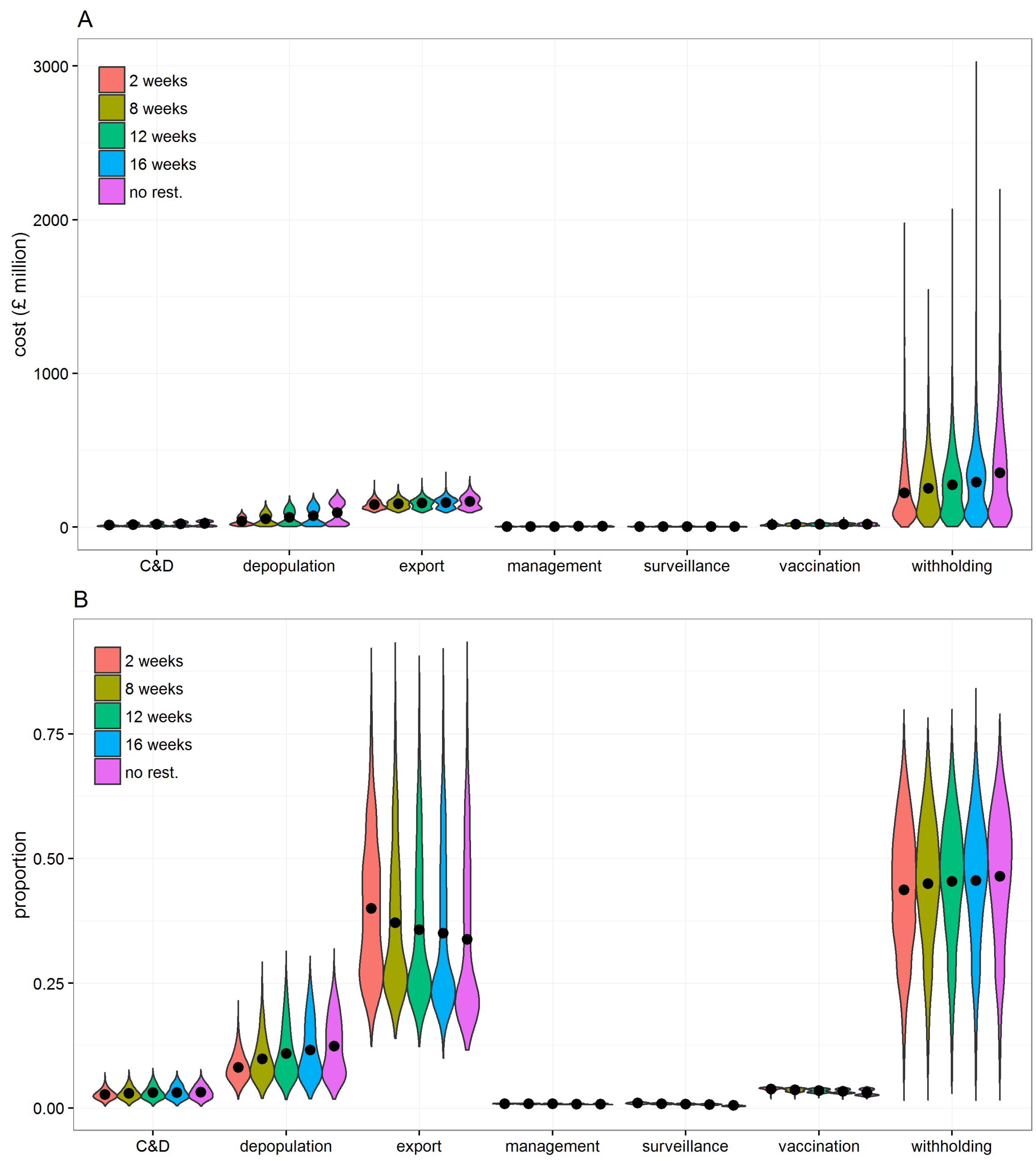

Figure S8: Impact of restocking delays on the contribution of each specific cost elements to the total direct economic cost when controlling large outbreaks of foot and mouth disease in Scotland. (A) Cost estimates (in million GBP) of each specific cost element. (B) Contribution (as a proportion) of each specific cost element to the total direct economic cost. Here, vaccination was implemented 14 days after detection of foot and mouth disease in Scotland and with an initial stock of 100,000 vaccine doses. If new stocks of vaccine doses have been ordered, restocking demand has been triggered when less than 10% of the initial vaccine stock remains. Considered cost elements are those related to (*i*) the cleaning and disinfection (C&D) of depopulated farms (including preliminary and secondary C&D), (*ii*) the depopulation of farms (including compensation and legal costs), (*iii*) the loss of export trade, (*iv*) managing disease control activities, (*v*) the implementation of surveillance activities during and post outbreak, (*vi*) the implementation of the vaccination-to-live strategy and the reduction in value of vaccinated animals, (*vii*) the implementation of a national movement ban (including the loss of trade and the reduction in value of withheld animals, losses due to the reduction of throughputs in Scottish abattoirs, and the worsening of animal welfare standard).

REFERENCES

[Dataset] BPEX (2011). Deadweight Average Pig Price (DAPP). http://www.bpex.org.uk/MarketIntelligence/data/DappEuroSpec.aspx. Accessed: 2012-11-15

Chénard, G., Miedema, K., Moonen, P., Schrijver, R. S., and Dekker, A. (2003). A solid-phase blocking elisa for detection of type o foot-and-mouth disease virus antibodies suitable for mass serology. *Journal of Virological Methods* 107, 89–98. doi:https://doi.org/10.1016/S0166-0934(02)00196-9

DEFRA (2011). *Contingency Plan for Exotic Notifiable Diseases of Animals* (London, UK: Animal Health and Veterinary Laboratories Agency)

[Dataset] HMRC (2011). Export Quarterly Trade Statistics from UK and region. https://www.uktradeinfo.com/Statistics/RTS/Pages/RTSArchive.aspx. Accessed: 2013-01-24

[Dataset] HMRC (2017). Overseas Trade Statistics by Commodity Code 2011-2016. https://www.uktradeinfo.com/Statistics/BuildYourOwnTables/Pages/Home.aspx. Accessed: 2017-02-27

Moxey, A. (2008). *Foot & mouth disease review 2007: Economic impact in Scotland*. Report, A report submitted by Pareto Consulting to the Scottish Government. doi:ISBN9780755917174

[Dataset] QMS (2011). Standard Quality Quotation (SQQ). http://www.qmscotland.co.uk/market. Accessed: 2012-11-15

Risk Solutions (2005). *Cost benefit analysis of foot and mouth disease controls* (Report D5100/R3 to the Department for Environment Food and Rural Affairs (DEFRA))
